# Editorial: Combining chemo/radio therapy and immunotherapy for cancers—perfect mix of old and new

**DOI:** 10.3389/fimmu.2022.1129623

**Published:** 2023-01-12

**Authors:** Clare Y. Slaney, Peng Luo, Feng-Ming Kong, Linlang Guo, Jian Zhang

**Affiliations:** ^1^ Cancer Immunology Program, Peter MacCallum Cancer Centre, Parkville, VIC, Australia; ^2^ Sir Peter MacCallum Department of Oncology, University of Melbourne, Parkville, VIC, Australia; ^3^ Department of Oncology, Zhujiang Hospital, Southern Medical University, Guangzhou, China; ^4^ Department of Clinical Oncology, Li Ka Shing Faculty of Medicine, University of Hong Kong, Pokfulam, Hong Kong SAR, China; ^5^ Department of Clinical Oncology, The University of Hong Kong-Shenzhen Hospital, Shenzhen, China; ^6^ Department of Pathology, Zhujiang Hospital, Southern Medical University, Guangzhou, China

**Keywords:** chemotherapy, radiotherapy, immunotherapy, cancer, combination therapy

We are honored to accept 48 articles published on the Research Topic of “*Combining chemo/radio therapy and immunotherapy for cancers—perfect mix of old and new*”.

While chemotherapy and radiotherapy are two classic treatments for tumors in the clinic, cancer immunotherapy has demonstrated its remarkable impact on clinical outcomes and revolutionized the field. Several types of immunotherapies, including immune checkpoint inhibitors (ICIs), antibodies, cancer vaccines, cytokines, adoptive cell transfer (ACT), especially chimeric antigen receptor (CAR) T cell therapy, have demonstrated durable clinical responses and obtained US Food and Drug Administration (FDA) approvals (1, 2). However, these immunotherapies only deliver clinical benefit to a small proportion of patients and are often associated with a high rate of toxicity. Meanwhile, chemotherapy and radiation can reshape the tumor immune microenvironment (TIME) and improve the immunotherapy treatment efficacy (3, 4). There is intense interest in combining chemo/radio therapy with immunotherapies to augment anti-tumor immune responses, reduce toxicity and improve treatment outcomes.

Radiotherapy is frequently used to treat cancers, due to its ability to cause DNA and mitochondrial damage in the tumor cells. In the clinic, radiotherapy is often applied before surgery as a debulking intervention, and sometimes used post-surgery to control residual disease. Some radiotherapy is also used in palliative settings to contain cancer symptoms. In recent years, radiotherapy has been identified to cause certain changes of the TIME, such as inducing the maturation of dendritic cells (DCs), the activation of T cells (5) and cause local cytokine release (Yu et al.). In addition, cancer cells that are irreversibly damaged by the radiotherapy release tumor antigens and emit immunostimulatory signals to support anti-cancer responses. Accumulating evidence supports that the ultimate efficacy of radiotherapy is dependent on the patient’s immune systems (6). Thus, it is logical to combine the treatments of radiotherapy and immunotherapies that aim to enhance the anti-cancer responses. In fact, growing evidence indicates that combining radiotherapy with anti-PD1 or anti-PD-L1 is associated with improved survival in both preclinical and clinical settings (Yu et al.; 6) .

It has been long understood that radiation causes abscopal responses of the tumor, which describes a phenomenon that when the local tumor is irradiated, the distal tumors are also reduced (7). There is clear evidence that the radiation induced abscopal effect is through immune dependent mechanisms (8). It is therefore rational to integrate immunotherapy with radiotherapy to induce a superior anti-cancer response at all sites of the disease. A number of articles in this issue propose new strategies to combine radiotherapy with immunotherapies. Sun et al., for example, used anti-PD-1 antibody, anlotinib and pegaspargase, followed by radiotherapy to treat localized natural killer/T cell lymphoma (NKTL) and demonstrated good efficacy and tolerance. Another promising strategy proposed by Qin et al., is to combine radiotherapy and CAR T cell adoptive transfer.

Chemotherapy has been used as the standard treatment in cancers for decades. Although chemotherapy is effective in the treatment of certain types of cancers, including cervical, ovarian testicular cancers, Hodgkin’s and non-Hodgkin’s lymphoma, it does not significantly improve overall survival of patients in other cancers (9). Instead, chemotherapy given as palliative therapy has low tumor specificity and is associated with numerous severe side effects.

Even though chemotherapy is historically considered immunosuppressive, recent work has clearly demonstrated that the impact of chemotherapy on the immune system depends largely on context (10) It is now accepted that certain chemotherapies can augment anti-tumor immune responses and synergize the clinical activity of the ICI treatments (9, 11). Chemotherapy can induce cancer cell death, enhance cancer neoantigen-presentation, disrupt tumor evasion of the immune recognition, sensitize the tumor cells to immune cell-mediated death, and change the immunosuppressive TIME, thus, augment anti-tumor immunity. In fact, the US FDA has approved several chemo-immunotherapy combinations for the treatment of cancers, especially in non-small cell lung cancer (NSCLC) patients (11). Several other chemo-immunotherapy combinations have also demonstrated promising results in a variety of cancers (11). There is intense interest to study how the cancer types and stages affect the action of chemotherapies on the anti-tumor immune responses.

In this issue, a number of studies supported the idea that chemo-immunotherapy combination has great potential in treating cancers of various types. Sue et al. reported treating a patient bearing a small cell neuroendocrine carcinoma (SCNEC) in the gynecologic tract with standard chemotherapy (paclitaxel + carboplatin) in combination with toripalimab (anti-PD-1) and achieved a complete response (CR). Until the date of publishing, the patient remained CR and had achieved 27 months of progression-free survival. This case report suggested the potential value of chemo-immunotherapy in a broader cancer range (Su et al.) than that approved by FDA and other authorities.

Of note, an extensively investigated area is the treatment induced changes of the TIME. The TIME that consists of many cell types, including immune cells, stroma, extracellular matrix and some soluble factors, is essential in determining the tumor response to therapies (Oliver et al.). Radiotherapy, certain chemotherapies and a number of other strategies have been reported to change the TIME to favor anti-tumor immunity. In this issue, a number of preclinical and clinical studies demonstrated the important role of TIME in determining treatment response and validated a number of biomarkers of the TIME predicting tumor responsiveness and toxicity (Zhang et al., Zeng et al., Liu et al., Wang et al.).

The optimal combination of chemo/radio therapy and immunotherapy is to generate the greatest synergy and minimize antagonym. With the understanding on the impact of therapies on TIME, tumors, and anti-tumor immunity; in depth exploration in prognostic and predictive markers and exploration of the underlying mechanisms; development of preclinical models that facilitate the research on human immunity and generation of bioinformatic tools for genetic/epigenetic/-omics studies, biomarker guided rational combinations will lead to more effective treatments for a wider range of cancers.

## Author contributions

CS wrote the manuscript. CS, PL, F-MK, LG and JZ contributed to conception and discussion. All authors contributed to manuscript revision, read, and approved the submitted version.
